# Embryo implantation is closely associated with dynamic expression of proprotein convertase 5/6 in the rabbit uterus

**DOI:** 10.1186/1477-7827-9-43

**Published:** 2011-04-06

**Authors:** Peter K Nicholls, Zhaogui Sun, Sophea Heng, Ying Li, Jian Wang, Guiying Nie

**Affiliations:** 1 Prince Henrys Institute of Medical Research, 246 Clayton Road, Clayton, Victoria 3168, Australia; 2 Department of Biochemistry and Molecular Biology, Monash University, Clayton, Victoria 3800, Australia; 3 Key Laboratory of Contraceptive Drugs and Devices of National Population and Family Planning Committee, Shanghai Institute of Planned Parenthood Research, 2140 Xie Tu Road, Shanghai 200032, China

## Abstract

**Background:**

Proprotein convertase 5/6 (PC5/6) is critical for embryo implantation in women, regulating both uterine epithelial receptivity and stromal cell decidualization. PC5/6 is likewise essential for implantation in mice, but involved only in decidualization. An alternative animal model is required to address the function of PC5/6 in the uterine epithelium. This study aimed to establish whether PC5/6 is associated with embryo implantation in rabbits.

**Methods:**

Virgin New-Zealand white rabbits aged 3-4 moths were mated with males of the same strain, or pseudo-pregnancy induced. After mating, uterine tissues were collected over a 10 day (d) period (n = 3 per time point) for RNA, protein and histological analyses to determine the temporal and spatial uterine expression pattern of PC5/6 during the initial stages of pregnancy or induced pseudo-pregnancy.

**Results:**

PC5/6 mRNA was up-regulated just prior to embryo attachment on d6, and the elevated expression was maintained throughout implantation on d6.5-10. Western analysis revealed a preferential up-regulation of PC5/6 in the implantation sites. Immunohistochemical analysis identified that both the amount and cellular localization of PC5/6 changed with increasing pregnancy stages. Before embryo attachment, PC5/6 was low and localised in the luminal and glandular epithelium. It increased on d6.5 in the basal glands and mucosal folds, and then strongly intensified on d7-10 in the multinucleated luminal symplasma and decidual cells at the site of embryo implantation. In contrast, the pseudo-pregnant uterus displayed relatively low and static PC6 mRNA expression throughout the 10 days, with no obvious changes in either PC5/6 level or cellular localization.

**Conclusions:**

These findings demonstrate that embryo implantation in the rabbit is closely associated with dynamic expression of uterine PC5/6, and that the rabbit may be an appropriate model to investigate the function of PC5/6 in the uterine epithelium during embryo attachment.

## Background

Embryo implantation is a complex process requiring a plethora of regulatory molecules, including proteases, cytokines and chemokines (reviewed in [1]). Failure of implantation accounts for approximately 75% of human pregnancy loss before 20 weeks of gestation, and is a major limiting factor in assisted reproduction [2,3]. Embryo implantation requires a healthy embryo and a receptive uterus that are synchronously developed. The uterus must undergo significant morphological and physiological changes to prepare for implantation [1]. In humans, two uterine events critical for implantation are the establishment of epithelial receptivity and stromal cell decidualization [4].

The proprotein convertases (PCs) are a family of 7 proteases that endoproteolytically cleave latent precursor proteins into their biologically active states (reviewed in [5]). PCs generate a large number of tissue-specific and functionally important bioactive proteins, and as such, they act as regulatory "master switches" by influencing cell proliferation, motility, adhesiveness and invasion [5]. PCs have been associated with a number of human diseases, including cancer, and are recognised as potentially important therapeutic targets [5-7].

PC5/6 is the only PC member that is associated with uterine remodelling and important for embryo implantation. PC5/6 is up-regulated in the uterus specifically at implantation in the mouse and human [8-10]. In mice, PC5/6 is up-regulated specifically in decidualizing stromal cells adjacent to the implanting embryo at the time of attachment and implantation [9]. PC5/6 is the only PC involved in decidualization in mice [10]. In women, PC5/6 is likewise dramatically increased during decidualization prior to implantation and intensifies with implantation establishment [11,12]. In contrast to the mouse, PC5/6 is also expressed in the uterine epithelium across the menstrual cycle in women peaking during the receptive phase, in addition to the decidual cells [12]. PC5/6 is the only PC tightly regulated in this manner [10,11,13]. Therefore, PC5/6 is likely to be involved in the establishment of epithelial receptivity as well as stromal cell decidualization in the human uterus.

Knockdown of PC5/6 prevents decidualization of human endometrial stromal cells in culture [14], and blocking PC5/6 uterine production inhibits decidualization and embryo implantation in mice [12], proving PC5/6 is critical for decidualization. The precise mechanisms of PC5/6 action in the uterine epithelium remain to be determined. Given that PC5/6 primarily regulates decidualization in the mouse, mice are a powerful *in vivo *model to investigate the functions of PC5/6 in decidualization. However, major uterine epithelial differences exist between rodents and humans, including the presentation of cell surface glycosylation and selectin ligands [15,16], and that PC5/6 is not expressed in the non-pregnant mouse uterine epithelium. Thus, an appropriate non-mouse animal model relevant to human implantation is required to investigate the *in vivo *function of PC5/6 in the uterine epithelium.

The rabbit is regarded as an excellent model to study the molecular events of embryo adhesion and attachment, as it employs a typical centric or fusion type of implantation in which the blastocyst adheres solely to the apical epithelial cells [17,18]. Given that rabbits are obligate ovulators, both pseudo-pregnancy and pregnancy can be precisely triggered, and ovulation (10 h after mating or hormonal treatment), embryo apposition and attachment (day 6.5-7) precisely timed. Furthermore, the rabbit uterus undergoes a dramatic transformation in the days after ovulation, such that days 1-4 and 4-7 are in some degree analogous to the proliferative and secretory phases of the primate menstrual cycle, respectively [19]. Rabbits have been used previously to investigate molecules associated with embryo attachment and implantation, including integrins, osteopontin, epidermal growth factors, mucin 1 and leukaemia inhibitory factor [18,20-23].

This study aimed to establish whether uterine PC5/6 is associated with embryo implantation in rabbits. The temporal and spatial expression pattern of PC5/6 was determined in the rabbit uterus during early pregnancy and pseudo-pregnancy. Real-time RT-PCR and Western blot analysis examined PC5/6 mRNA and protein, respectively. The cellular localization and expression pattern were also determined by immunohistochemistry. The results demonstrate that uterine PC5/6 is dynamically regulated in the rabbit during implantation, especially in the epithelium, suggesting that the rabbit will provide a useful animal model to examine the roles of PC5/6 in uterine epithelium for embryo attachment.

## Methods

### Animals

New Zealand White rabbits aged 3-4 months (2.6-3.2 kg) were maintained at Shanghai Institute of Planned Parenthood Research Animal Facility under controlled conditions (14 h light-10 h dark cycle, 22°C) with free access to food and water. All experimentation was performed in compliance with the laboratory animal care protocols approved by the Institutional Animal Care Committee of the Shanghai Institute of Planned Parenthood Research.

Virgin female rabbits were mated with males of the same strain, with the time of mating designated as day (d) 0 of pregnancy. Animals were euthanased at various time points following mating (d0, 1, 3, 5, 6, 6.5, 7, 8 and 10, n = 3 each) by injecting 20 ml air into the auricular veins. To induce pseudo-pregnancy, rabbits received an i.m. injection of 20 IU pregnant mare serum gonadotrophin, and 72 h later, an i.m. injection of 0.8 μg of gonadotrophin-releasing hormone (GnRH). Ovulation was confirmed in each animal by the presence of the corpus luteum in each ovary.

Uterine tissues were collected at each time point, and either snap-frozen in liquid nitrogen for RNA and protein analysis, or fixed in 4% paraformaldehyde (in 0·1 M sodium phosphate buffer pH 7.4) for 48 h followed by paraffin-embedding for immunohistochemistry. From the pregnant rabbits, implantation (Imp) and inter-implantation (Inter) site tissues were collected separately for RNA (d6.5 onwards, n = 3) and protein (d7.5 onwards) analysis.

### RNA isolation and real-time PCR analysis

Total RNA was extracted using Trizol reagent (Invitrogen, Carlsbad, CA) as per the manufacturer's instructions. Contaminating DNA was removed using a DNAse free kit (Ambion, Austin, TX). RNA quality and quantity was assessed by optical densitrometry (Nanodrop, ThermoFisher Scientific, Waltham MA). Reverse transcription was performed using 500 ng RNA with SuperScript VILO cDNA synthesis kit (C#11754, Invitrogen, Carlsbad, CA), and the resulting products were snap-frozen and stored at -80°C.

Quantitative real-time PCR analysis was performed using the Corbett Rotorgene 3000 (Corbett Lifesciences, Sydney, Australia) with the FastStart DNA Master SYBR-Green 1 system (Roche Applied Sciences, Basel, Switzerland). Oligonucleotide sequences and conditions for PC5/6 and 18S amplicons are shown in Table 1. For quantification, a reference cDNA was prepared from a rabbit uterine tissue for quality control and standard. After 38 cycles, a melting curve analysis was performed to monitor PCR product purity. In initial experiments, amplification of a single PCR product was confirmed by agarose gel electrophoresis and DNA sequencing. Data was normalised to 18S and the mean from each triplicate samples from three animals were compared.

**Table 1:** Primer sequences and conditions used for quantitative PCR analysis

Gene Name	Primer Sequence (5'-3')	Size (bp)	Anneal temp (°C)	Extension time (s)	Mg2+ (mM)
PC5/6	F: TCTGACCTGGAGAGACGTAC R: TGTCTTGATATGTCGATCTG	221	55	9	3.0
18S	F: CGGCTACCACATCCAAGGAA R: GCTGGAATTACCGCGGCT	187	64	8	3.0

F, Forward; R, Reverse

### Western blot analysis

Frozen uterine tissues were thawed and homogenized using a glass homogenizer in extraction buffer, containing 6.0% (w/v) SDS, 0·14 mol/L Tris-HCl (pH 6.8), 22.4% (v/v) glycerol, soybean trypsin inhibitor (10 μg/mL, Sigma, St. Louis, MO), PMSF (1 mmol/L, Sigma,), leupeptin and aprotinin (10 μg/mL each, Amresco, Solon, OH). The homogenates were centrifuged (14,000*g*/10 min) at 4 °C, the supernatant containing proteins was collected and protein concentrations were determined using the Bradford assay. Proteins (50 μg) were subjected to reducing 12% sodium dodecyl sulfate-polyacrylamide gel electrophoresis (SDS-PAGE) and transferred onto nitrocellulose membranes (ImmobilonTM-NC, Millipore Corporation, Billerica, MA, USA). The membrane was blocked for 1 h in 5% skim milk in 0.2% Tween-20 (Sigma, St. Louis, MO, USA), and probed overnight at 4°C with a previously characterized affinity purified polyclonal sheep anti-human PC5/6 antibody (25 μg/ml in blocking solution, Nie et al., 2005b). The membrane was incubated for 1 h with HRP-conjugated donkey anti-sheep IgG (Milllipore, Billerica, MA, 1:10,000 dilution in blocking solution) at room temperature, and the signal visualized by chemiluminescent detection (Thermo Scientific, Rockford, IN). The membrane was subsequently stripped and re-probed with a β-actin antibody (Cell Signaling, Danvers, MA).

### Immunohistochemical analysis

Immunohistochemistry was performed using the sheep PC5/6 antibody (n = 3 sites per animals, n = 3 animals for each time point) as previously described [8]. Briefly, formalin-fixed paraffin sections (5 μm) were de-waxed in histosol (Sigma), rehydrated, and subjected to antigen retrieval by microwave (5 min/700W followed by 5 min/140 W) in 0·01 mol/L sodium citrate buffer (pH 6.0). Endogenous peroxidase activity was quenched with 6% H_2_O_2 _(diluted 1:1 v/v in 100% methanol) for 10 min at room temperature. Nonspecific binding was blocked by incubation with 15% normal rabbit serum and 5% fetal calf serum in TBS containing 0.1% Tween-20. Primary antibody was applied (10 μg/mL) at 37 °C for 2 h. Following washing in TBS, slides were incubated with biotinylated rabbit anti-sheep IgG (1:200 dilution, Vector Laboratories, Burlingame, CA) for 30 min at room temperature. Signal was amplified using Vectastain ABC amplification (Vector Laboratories) for 30 min and positive localization visualized using the peroxidise substrate 3, 3'-diaminobenzidine (DAB, Dako, Kingsgrove, Australia) which produced a brown precipitate. Tissue sections were counterstained with Harris' hematoxylin (Sigma Chemicals, St Louis, MO), dehydrated, and mounted with DPX (ProSciTech, Thuringowa, Australia). Negative controls were included for each section, where pre-immune sheep IgG was substituted for the primary antibody at a matching concentration. The localization of staining was assessed by two investigators who noted expression patterns, for which representative images were recorded.

### Statistics

Unless otherwise noted, all measurements were on samples from three separate animals, from which the mean and SEM were calculated. All statistics were performed using SigmaStat version 3.5 (Systat Software, Inc., San Jose, CA). Homogeneity of variance was assessed for all groups by normality and equal variance tests. Experiments in which variation followed a normal distribution were assessed using a one-way ANOVA, followed by the Student-Newman-Keuls post hoc multiple group comparisons test for significance. P value of < 0.05 was used as a cut-off for statistical significance.

## Results

### RT-PCR analysis of PC5/6 mRNA in the rabbit uterus during early pregnancy

**Figure 1:**
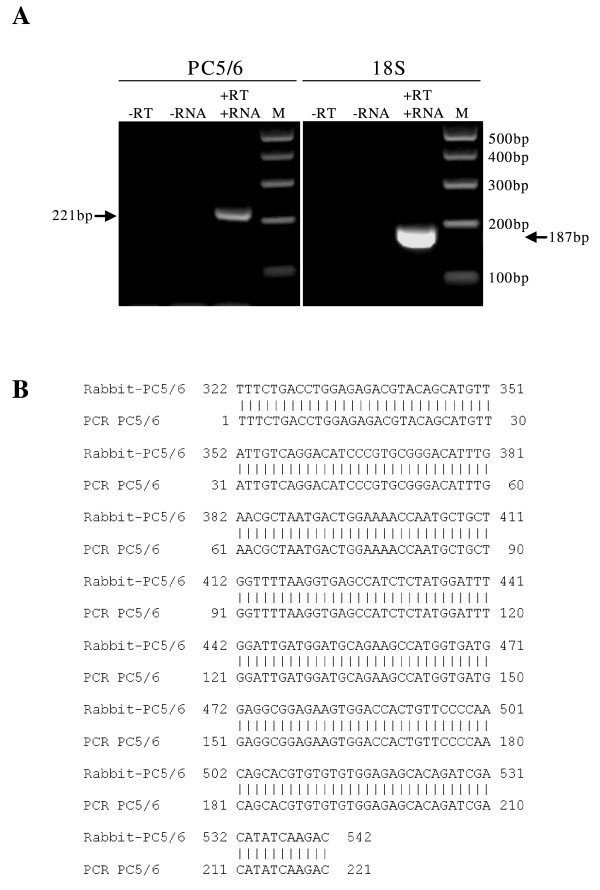
**RT-PCR confirmation of PC5/6 mRNA expression in the rabbit uterus**. **(A) **Agarose gel electrophoresis of RT-PCR products of PC5/6 (220 bp) and 18S (187 bp) respectively. Two negative controls are: -RT, no reverse transcription enzyme in RT reaction; -RNA, no RNA in RT reaction. +RT+RNA, RT reaction contained RNA and reverse transcription enzyme. M, DNA marker. **(B) **Comparison between sequences of rabbit PC5/6 mRNA (Rabbit-PC5/6) and RT-PCR amplified uterine PC5/6 product (PCR-PC5/6).

Primers specific to the rabbit PC5/6 mRNA (both A and B isoforms) coding region were designed and conditions for RT-PCR optimized (Table 1). Amplification of 18S was used for data normalization. A single band of expected size was amplified for PC5/6 (221 bp) and 18S (187 bp) from rabbit uterine RNA (Figure 1A). Melting curve analysis validated each band as a single DNA product (data not shown). No amplification occurred in the negative controls when either the reverse transcriptase or RNA was omitted (Figure 1A). The PC5/6 product was sequenced and found to be 100% homologous to the rabbit PC5/6 mRNA (Figure 1B), confirming specificity. This established that PC5/6 mRNA was expressed in the rabbit uterus, and that the newly designed primers were specific and suitable for analysing PC5/6 mRNA in the rabbit.

**Figure 2:**
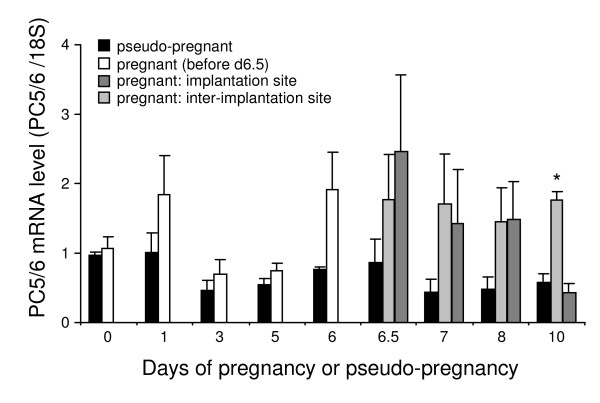
**Real-time RT-PCR analysis of PC5/6 mRNA expression in the rabbit uterus between d0 and d10 in pregnant and pseudo-pregnant animals**. For pregnant uterus from d6.5 onwards, implantation and inter-implantation sites were collected separately. Data are mean + SEM, n = 3 animals, * P < 0.001 (inter-implantation site vs implantation site and pseudo-pregnant uterus on the same day).

The expression of PC5/6 mRNA in the rabbit uterus during early pregnancy and pseudo-pregnancy was analysed by quantitative RT-PCR. The first 10 days of pregnancy including the period of embryo attachment (d6.5-7) and implantation (d8-10) were examined. For each real-time run, the cycle threshold was determined for replicates of each sample and compared to the linear standard curve, then normalised to 18S expression. The mean and SEM were then determined for each triplicate of animals and are presented in Figure 2. Pseudo-pregnant rabbits showed no significant changes in PC5/6 expression across the 10 days following GnRH administration. In contrast, the pregnant uterus displayed a dynamic pattern of PC5/6 expression during the same time-course (Figure 2). PC5/6 mRNA levels were relatively static during the initial 5 days (except a non-significant increase on d1), but a sustained trend of increased expression in pregnant tissues commenced from d6 (immediately prior to implantation, 1.9 fold greater than d0 level). On d6.5, corresponding to the time of initial embryo attachment, the mean level at the implantation and inter-implantation sites compared to d0 was 2.34 and 1.82 fold respectively. These increased levels were maintained throughout d6.5-8 at both the implantation or inter-implantation sites, and on d10 at the inter-implantation sites (Figure 2).

When pregnant and pseudo-pregnant animals were compared at the equivalent time points, no significant difference in PC5/6 mRNA levels was found during d0-5 (Figure 2). However, from d6 onwards the expression of PC5/6 was found to be higher in the pregnant uterus. On d6, the pregnant uterus was 2.5 fold of the pseudo-pregnant counterpart (Figure 2). On d6.5 (the putative time of embryo attachment), the fold differences were 2.9 and 2.1 for the implantation and inter-implantation sites compared to the pseudo-pregnant uterus, respectively. This trend was maintained between d6.5-8, with a peak difference between the pregnant and pseudo-pregnant uteri on d7 of 3.2- and 3.9-fold in the implantation and inter-implantation sites, respectively (Figure 2). On d10, PC5/6 levels in the inter-implantation sites were 3.0 fold greater than the pseudo-pregnant value (p < 0.001, Figure 2). This data reveals that PC5/6 expression is increased in the rabbit uterus at the time of embryo attachment and implantation, and that this dynamic regulation was specific to pregnancy.

### Western blot analysis of PC5/6 protein in the rabbit uterus during implantation

**Figure 3:**
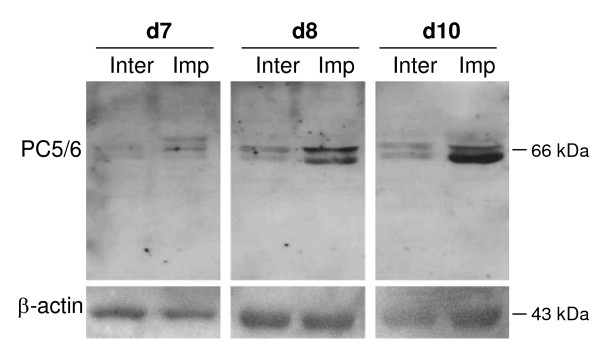
**Western blot analysis of PC5/6 protein in the pregnant rabbit uterus**. Total protein extracts from inter-implantation (Inter) and implantation (Imp) sites on pregnant d7, 8 and 10 were analysed for PC5/6 (upper panel) and β-actin (lower panel) respectively.

The presence of PC5/6 protein in the rabbit uterus was established by Western blot analysis. Total proteins isolated from implantation and inter-implantation sites on d7-10 were probed with the PC5/6 antibody. The membrane was then re-probed for β-actin as a loading control (Figure 3). Two bands at approximately 66 kDa were detected for PC5/6 in all samples, consistent with previous findings in the mouse [8,24]. A much higher level of both bands of PC5/6 protein was detected in d8 and d10 isolates than d7 (Figure 3) Furthermore, the expression of PC5/6 protein was more intense in the implantation than the inter-implantation sites at each time point (Figure 3). The prevalence of truncated forms of PC5/6 may represent an additional regulatory mechanism in the rabbit uterus. These data confirmed the specificity of the PC5/6 antibody in the rabbit uterus, and demonstrated that PC5/6 protein was increased during embryo implantation.

### Immunolocalization of PC5/6 protein in the rabbit uterus during early pregnancy

**Figure 4:**
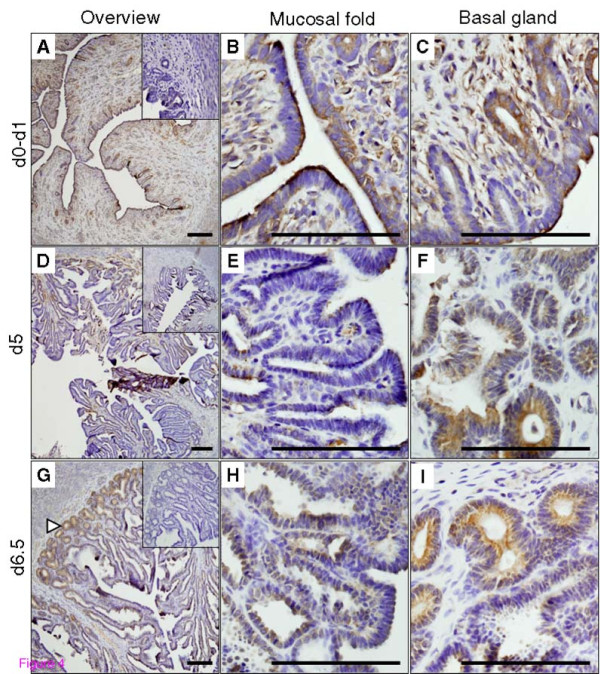
**Immunolocalization of PC5/6 protein in the rabbit uterus during pregnancy d0-6.5**. Representative microphotographs are shown for pregnant d0-1 (A, B and C), d5 (D, E and F) and d6.5 (G, H and I). For each time point, panels depict an overview (A, D and G), endometrial mucosal folds (B, E and H) and basal glands (D, F, I). Arrowhead in G demonstrates increased basal glandular staining at the time of embryo attachment. Insets in A, D and G are negative controls. Scale bar = 25 μm.

The cellular localization and expression pattern of PC5/6 protein in the rabbit uterus across early pregnancy and pseudo-pregnancy was then determined by immunohistochemistry using formalin-fixed tissues and the PC5/6 antibody previously validated by Western blot. In the pregnant uterus, between d0-1 of pregnancy (Figure 4A-C), endometrial stroma and luminal epithelium demonstrated low levels of immuno-reactive PC5/6. The basal regions of luminal epithelium folds were also PC5/6 positive (Figure 4B-C), and to a lesser extent, positivity was also observed in the endothelial cells (Figure 4B-C). Between d3-5 (Figure 4D-F), the endometrial cells proliferate extensively, leading to the formation of mucosal folds. During this period, little immuno-reactive PC5/6 was detected in the endometrial folds (Figure 4E). In contrast, the deep glands located in the crypts against the myometrium were clearly positive (Figure 4F). At the time of embryo attachment on day 6.5 (Figure 4G-I), PC5/6 positivity in the deep basal glands became more prominent (Figure 4I). At this point of uterine remodelling, the mucosal folds were further extended compared with the early stages and were positive for PC5/6 (Figure 4G-H).

**Figure 5:**
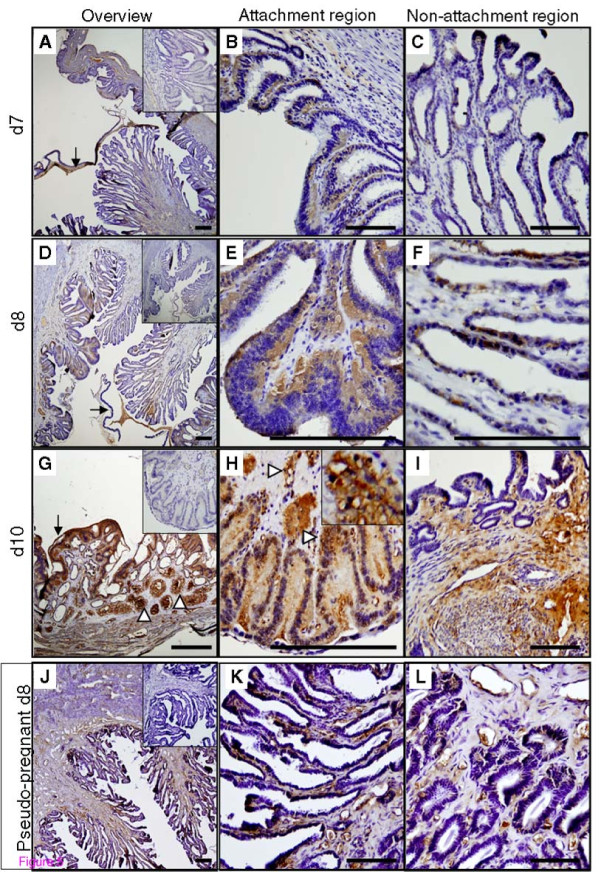
**Immunolocalization of PC5/6 protein in the rabbit uterus on pregnant d7-10 and pseudo-pregnant d8**. Representative microphotographs are shown for pregnant uterus on d7 (A, B and C), d8 (D, E and F) and d10 (G, H and I), and pseudo-pregnant uterus on d8 (J, K and L). For pregnant uterus, panels depict an overview (A, D, G), attachment region (B, E and H) and non-attachment region (D, F and I) for each time point. Arrows in A, D and G highlight the embryonic tissues. Arrowheads in H show decidual cells; the insert shows a higher magnification. For pseudo-pregnant uterus, an overview (J), mucosal folds (K) and deep basal glands (I) are shown. Inserts in A, D, G and J are negative controls. Scale bar = 25 μm.

PC5/6 levels and localization transformed significantly during the time of embryo attachment and implantation (Figure 5). Immediately after initial embryo attachment on d7 (Figure 5A-C), PC5/6 was differentially expressed between the attachment (antimesometrial side) and non-attachment regions (mesometrial side). Whilst the mesometrial folds maintained PC5/6 immuno-negativity (Figure 5C), the antimesometrial luminal epithelium closely apposed to the blastocyst, now fusing, becoming multinucleated and transforming to a structure termed symplasma, became PC5/6 immuno-positive (Figure 5B). This pattern of localization persisted with gestation, and the intensity of immuno-reactive PC5/6 increased progressively (Figure 5). By d8 (Figure 5D-F), PC5/6 staining was much stronger than on d7 in the symplasma (Figure 5E). Noticeably, the staining was polarized within the multinucleated symplasma, with a prominent accumulation of PC5/6 protein at the basal surface (Figure 5E). On d10, very strong PC5/6 staining was detected at the site of embryo implantation (Figure 5G-5H). Strong immuno-reactive PC5/6 was seen in the multinucleated luminal epithelium cells (Figure 5H), which now appeared to be evenly distributed, rather than polarised. In addition, strong PC5/6 staining was evident in the peri-vascular decidual cells (Figure 5H, arrow head and insert). The luminal epithelium and glandular epithelium away from the implantation site maintained PC5/6 negativity (Figure 5I), but positivity was evident in the stroma of this region.

The uteri of pseudo-pregnant rabbits (d0-d10) were also examined by immunohistochemistry. The morphological changes during pseudo-pregnant d0-5 resembled those of pregnant rabbits on the equivalent days (data not shown). Likewise, uterine PC5/6 expression and localization were similar between pregnant and pseudo-pregnant rabbits during this period (d0-5, data not shown). However, unlike the pregnant uterus, the pseudo-pregnant uterus showed no further uterine remodelling with increasing gestation after d5. While a significant increase in PC5/6 staining was detected specifically at implantation sites in the pregnant uterus, no such changes were observed in the epithelial cells during pseudo-pregnancy, and PC5/6 levels and localization in the pseudo-pregnant uterus on d8-10 were similar to that of d5 pregnant uterus. Example images of PC5/6 immunolocalization in d8 pseudo-pregnant uterus are shown in Figure 5J-L.

Collectively, this data establishes that PC5/6 protein is dynamically regulated during early pregnancy in the rabbit uterus. PC5/6 was up-regulated in the endometrial deep glands just prior to embryo attachment. PC5/6 localization then strongly intensified in the multinucleated luminal symplasma and decidual cells at the site of embryo attachment during the time of implantation. PC5/6 up-regulation was both pregnancy, and implantation-site specific.

## Discussion

This study established that dynamic expression of PC5/6 in the rabbit uterus is closely associated with embryo implantation and pregnancy. Uterine PC5/6 mRNA was up-regulated just prior to embryo attachment on d6 of pregnancy, which was maintained throughout implantation between d6.5-10. In contrast, no such changes were observed in the pseudo-pregnant rabbits as they displayed a low and static PC5/6 mRNA level throughout the 10 days of pseudo-pregnancy. Western blot analysis revealed a preferential up-regulation of PC5/6 protein at embryo implantation sites. Immunohistochemical analysis confirmed this pattern and illustrated that PC5/6 protein level and cellular localization changed according to pregnancy stage. Prior to embryo attachment, PC5/6 expression is low and localised primarily in the luminal epithelium and endometrial glands. Subsequently, PC5/6 expression increased in the deep basal glands and mucosal folds just prior to embryo attachment, and then strongly intensified in the multinucleated luminal symplasma and decidual cells at the site of embryo implantation from d7-10. In contrast, no obvious change in either PC5/6 expression or cellular localization occurred across 10 days of pseudo-pregnancy. This study thus demonstrates that uterine PC5/6 up-regulation is pregnancy-stage, and implantation-site specific.

The overall pattern and cellular localization of PC5/6 in the rabbit uterus during early pregnancy is more analogous to that of the human than the mouse. In both the human and mouse uterus, PC5/6 is specifically expressed in the decidual cells in the stromal compartment, but glandular and luminal epithelial expression is only observed in the human uterus [8,9]. This study identified uterine PC5/6 expression in both the epithelium and decidual cells in the rabbit, as in the human, establishing the basis for using the rabbit as a model system to investigate the function of PC5/6 in the uterine epithelium. It is worth noting that in the rabbit uterus, even though the degree of stromal decidualization is minimal, decidualizing cells at the implantation site clearly expressed PC5/6, making decidual PC5/6 expression a common feature among mice, rabbits and humans.

Rabbit implantation employs a typical fusion type of implantation in which the blastocyst adheres solely to the luminal epithelial cells [17]. Distinct up-regulation and immunolocalization of PC5/6 in the uterine epithelium at the site of implantation from the very early stages of attachment strongly indicates a role for epithelial PC5/6 in blastocyst adhesion. The apical surface of the endometrial epithelium is constitutively non-adhesive (thus non-receptive) except during implantation. Conversion from a non-receptive to receptive state requires the epithelial cell acquisition of surface components required for receptor-ligand interactions, and alterations/modifications to adhesion molecules. During receptivity, the apical glycocalyx is reduced, and the normally abundant apical microvilli gradually retract, generating a flattened epithelial surface for blastocyst adhesion [25]. PC5/6 is known to endo-proteolytically activate a number of important adhesion molecules including neural adhesion protein-L1 [26] and integrins [27,28]. Of note, the intergrins are among the best characterized markers of uterine receptivity in a number of species including the rabbit [25]. Indeed, functional studies have confirmed that the α_v_β_3 _integrin complex is important for implantation in the rabbit [22]. It is thus possible that PC5/6 mediates integrin activation in the epithelium for embryo attachment and implantation in the rabbit.

PC5/6 can interact with heparin sulfate proteoglycans (HSPGs), thereby recruiting itself to the cell surface to activate a number of HSPG-binding proteins at the cell periphery or in the extracellular space [29,30]. HSPGs and HSPG-binding proteins participate in cell adhesion during implantation [25]. One such HSPG molecule is heparin-binding EGF-like growth factor (HB-EGF), whose expression is dramatically increased during pregnancy and restricted to implantation sites [31]. Since PC5/6 has been suggested activate HB-EGF on the cell surface [29,30], it is likely that PC5/6 regulates adhesion molecules and other cell surface proteins, in the endometrial epithelium during embryo attachment.

Pseudo-pregnancy is commonly used to investigate whether the expression of a particular gene is hormone-dependent or requires the presence of a blastocyst. This study suggests that PC5/6 regulation in the rabbit uterus is complex and pregnancy-specific. Prior to embryo attachment, PC5/6 expression and localization was comparable between pregnant and pseudo-pregnant uteri. Immediately prior to initial embryo attachment, PC5/6 mRNA was specifically up-regulated in the pregnant uterus, and PC5/6 protein was distinctly localised at the site of embryo attachment. This pattern of expression is consistent in principle with mouse and human studies. In the mouse, PC5/6 is up-regulated only at the site of embryo attachment, and this expression intensifies with increasing implantation stages [9]. In the human, PC5/6 expression in the decidual cells increases with implantation establishment [8]. Further studies are needed to address the contribution of the blastocyst and/or the blastocyst-endometrium interaction to promote PC5/6 expression. Future studies are required to determine the importance of PC5/6 in implantation in the rabbit, by direct inhibition of PC5/6 production or activity in the rabbit uterus *in vivo*.

## Conclusions

In summary, our studies have demonstrated that rabbit uterine PC5/6 expression is intimately associated with pregnancy and embryo implantation. Uterine PC5/6 expression is dynamically regulated in pregnancy, and distinctively expressed at the site of embryo attachment, supporting an essential role for this molecule in embryo attachment and implantation. Therefore, this study has established that the rabbit will be a valuable *in vivo *model to investigate the physiological role of PC5/6 in the uterine epithelium for embryo attachment and implantation.

## Abbreviations

PC5/6: pro-protein convertase 5/6; Inter: inter-implantation site; Imp: implantation site.

## Competing interests

The authors declare that they have no competing interests.

## Authors' contributions

PKN carried out the RNA and immunohistological analyses, and assisted in the drafting of the manuscript. ZS carried out the animal studies and assisted in the tissue collection SH carried out the protein analyses YL assisted with the RNA and immunohistological analyses. JW supervised the animal studies and assisted in the tissue collection GN conceived and designed the project, supervised the overall project and prepared the manuscript. All authors read and approved the final manuscript.

## Author Information

Correspondence to: Guiying Nie, Ph.D., Prince Henry's Institute of Medical Research, 246 Clayton Road, Clayton, Victoria 3168, Australia. E-mail: http://guiying.nie@princehenrys.org. Phone: +61 3 9594 4380. Fax: +61 3 9594 6125
